# Meta-analysis of segmentectomy versus lobectomy for radiologically pure solid or solid-dominant stage IA non-small cell lung cancer

**DOI:** 10.1186/s13019-019-0996-6

**Published:** 2019-11-13

**Authors:** Sunyin Rao, Lianhua Ye, Li Min, Guangqiang Zhao, Ya Chen, Yunchao Huang, Jichen Yang, Shouyong Xiao, Run Cao

**Affiliations:** 1 Department of Thoracic Surgery, The Third Affiliated Hospital of Kunming Medical University, Kunming, China; 2 Department of Respiratory Medicine, The Second Affiliated Hospital of Kunming Medical University, Kunming, China

**Keywords:** Non-small cell lung cancer, Segmentectomy, Lobectomy, Prognosis, Pure solid or solid-dominant, Meta-analysis

## Abstract

**Objective:**

Whether segmentectomy can be used to treat radiologically determined pure solid or solid-dominant lung cancer remains controversial owing to the invasive pathologic characteristics of these tumors despite their small size. This meta-analysis compared the oncologic outcomes after lobectomy and segmentectomy regarding relapse-free survival (RFS) and overall survival (OS) in patients with radiologically determined pure solid or solid-dominant clinical stage IA non-small cell lung cancer (NSCLC).

**Methods:**

A literature search was performed in the MEDLINE, EMBASE, and Cochrane Central databases for information from the date of database inception to March 2019. Studies were selected according to predefined eligibility criteria. The hazard ratio (HR) and associated 95% confidence interval (CI) were extracted or calculated as the outcome measure for data combining.

**Results:**

Seven eligible studies published between 2014 and 2018 enrolling 1428 patients were included in the current meta-analysis. Compared with lobectomy, segmentectomy had a significant benefit on the RFS of radiologically determined pure solid or solid-dominant clinical stage IA NSCLC patients (combined HR: 1.46; 95% CI, 1.05–2.03; *P* = 0.024) and there were no significant differences on the OS of these patients (HR: 1.52; 95% CI, 0.95–2.43; *P* = 0.08).

**Conclusions:**

Segmentectomy leads to lower survival than lobectomy for clinical stage IA NSCLC patients with radiologically determined pure solid or solid-dominant tumors. Moreover, applying lobectomy to clinical stage IA NSCLC patients with radiologically determined pure solid or solid-dominant tumors (≤2 cm) could lead to an even bigger survival advantage. However, there are some limitations in the present study, and more evidence is needed to support the conclusion.

## Introduction

The development and widespread use of computed tomography (CT) for lung cancer screening has enabled an increasing ability to detect small lung nodules [1]. Segmentectomy has gained increasing attention because this method can preserve more lung tissue and better improve the short-term outcomes than lobectomy [2–4].

At present, it is acknowledged that GGO (Ground glass opacity)-dominant early-stage NSCLC is associated with a good prognosis [5–10] and can be treated with sublobar resection (including segmentectomy or wedge resection), because these tumors are minimally invasive [9–11]. On the other hand, radiologically determined solid-dominant NSCLCs represent more malignant potential, such as vessel invasiveness and lymph node metastasis, compared with GGO-dominant tumors [12]. Moreover, pure solid tumors are associated with worse survival outcomes compared with part-solid tumors, even if the tumors are less than 2 cm in size [13]. In addition, postoperative nodal involvement was pathologically found in approximately 16–26% of lung cancer patients with radiographically determined tumors smaller than 2 cm [14, 15]. Two recent studies [16, 17] suggested that the RFS and OS were lower after segmentectomy than after lobectomy for radiologically determined solid clinical IA (≤2 cm) NSCLC, and segmentectomy was considered an independent risk factor for poor locoregional recurrence-free survival in a multivariate analysis. These results indicate that intentional segmentectomy may not be applicable for small radiographically determined invasive NSCLC. In contrast, many recent studies [18–22] have suggested some different results, revealing that segmentectomy for radiologically pure solid or solid-dominant stage IA NSCLC may have similar long-term effects to lobectomy. Hence, the use of segmentectomy for solid-dominant or pure solid tumors as a radical procedure is controversial.

In this context, a meta-analysis was performed by collecting current comparative studies to evaluate and compare the prognoses after segmentectomy with those after lobectomy in patients with radiologically determined solid-dominant or pure solid clinical stage IA NSCLC. The research results are expected to provide a reference for clinical decision making regarding the management of solid nodules.

## Methods

### Search strategy and eligibility criteria

We performed a search in MEDLINE, EMBASE and Cochrane Central for studies published before March 2019 using the following terms:(“lung” OR “pulmonary”) and (“neoplasm” OR “cancer” OR “carcinoma”) and (“lobectomy” OR “segmentectomy” OR “sublobar resection” OR “limited resection”) and (“recurrence” OR “prognosis” OR “survival”). The references of relevant articles were also scanned to identify other potentially eligible reports. Two authors worked independently to extract the general information and patient clinical characteristics from each eligible report.

All retrieved articles were then further assessed by the inclusion and exclusion criteria described. The inclusion criteria were as follows: 1) comparison of relapse-free survival (RFS) or overall survival (OS) between lobectomy and segmentectomy; 2) study subjects were limited to clinical Stage IA patients with a solid-dominant or pure-solid appearance on thin-section computed tomography, and “solid-dominant” and “pure solid” were defined as 0.5 ≤ consolidation/tumor ratio (CTR) < 1.0 and “CTR = 1”, respectively; 3) the baseline characteristics of patients treated with the two operative techniques were sufficiently balanced; and 4) if the enrolled patients were from the same institutions and the same period, the most recently published data would be included in the study. The exclusion criteria were as follows: 1) non-English articles; 2) letters, editorials, case reports, and reviews; 3) follow-up period less than 5 years; and 4) unavailable full text of the study.

### Extraction of effect size and statistical analysis

The risk ratio (HR) and associated 95% confidence interval (CI) were extracted from the literature for meta- analysis of RFS or OS. If the HR was not reported directly, then the value was estimated with methods reported in the literature [23–25]. Chi-square and I^2^ tests were used to assess heterogeneity. If there was no significant heterogeneity among the studies (*P* > 0.1, I^2^ < 50%), the fixed-effect model was used for combined analysis. If heterogeneity existed among the studies (*P* < 0.1, I^2^ > 50%), the causes of heterogeneity were explored and the random effect model was applied to pool the heterogeneous studies. Publication bias was assessed by Begg’s funnel plot and Egger’s test. The protocol for this systematic review was registered on PROSPERO and can be accessed at http://www.crd.york.ac.uk/prospero/ display_record.asp? ID = CRD42019129023. Kaplan-Meier curves were read by Origin version 2019(www.originlab.com). Calculations were performed using Stata version 15.1 (StatCorp, College Station, TX, USA). All *P* values were two tailed, and statistical significance was set as *P* < 0.05.

## Results

**Fig. 1:**
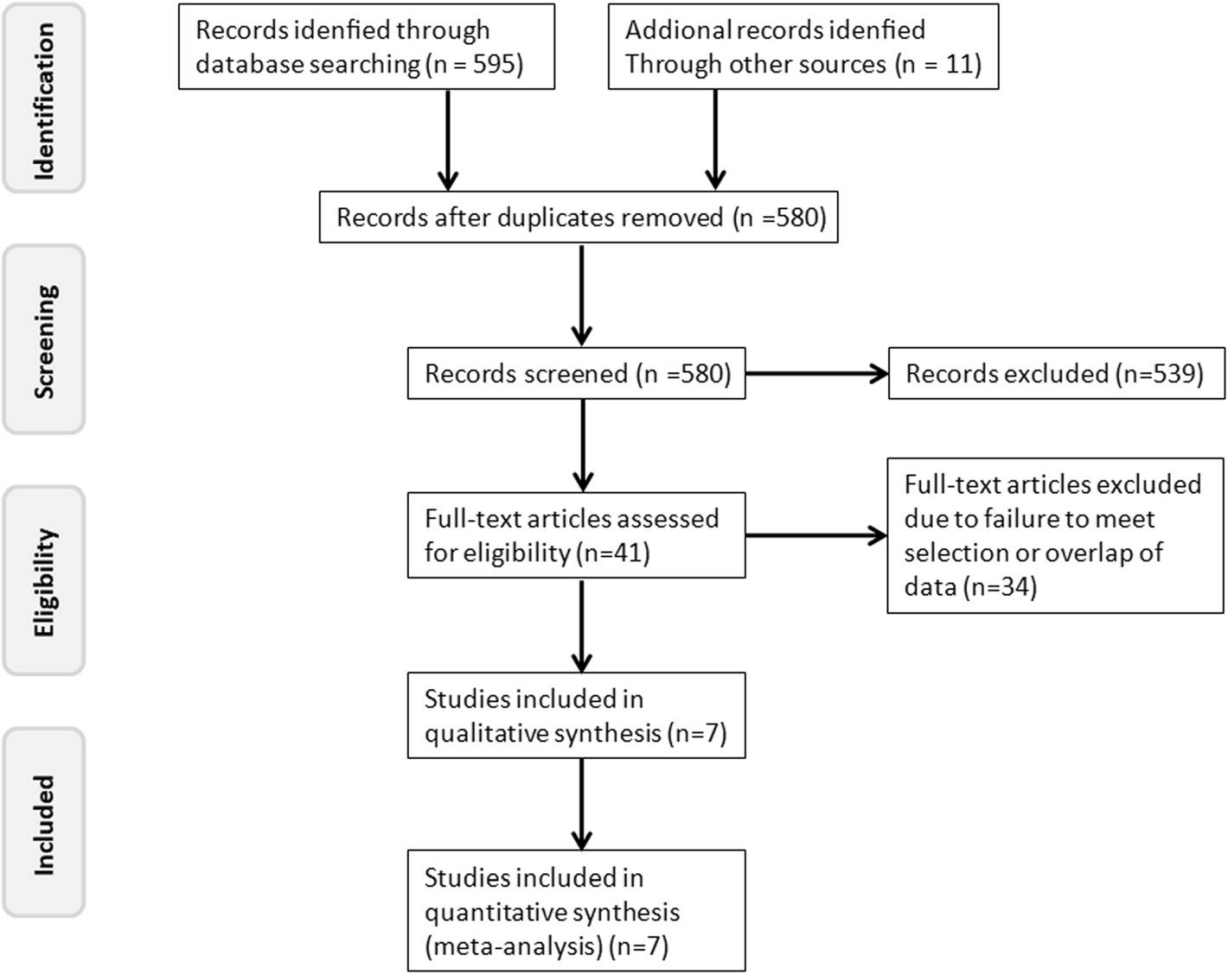
Flow diagram for study selection

We obtained a total of 606 papers from the searches. According to the inclusion and exclusion criteria, a total of 7 studies were included in the meta-analysis. The screening process for the studies is shown in Fig. 1, and the characteristics of the included studies are summarized in Table 1. All studies were published in recent years (between 2014 and 2018). No randomized controlled trials were found, and all included studies were retrospective studies. The total number of patients was 1428; of these patients, 987 were treated with lobectomy, and 441 received segmentectomy. In the analysis, the lobectomy group was chosen as the reference.

**Table 1:** Characteristics of included articles for meta-analysis

Authors	year	Study type	Clinical Stage	Tumor size	Segmentectomy(n)	Lobectomy(n)	consolidation/tumor ratio (CTR)
Nishio [16]	2016	Retrospective	IA	≤2 cm	118	72	>0.5
Hattori(1) [18]	2015	Retrospective	IA	2<T ≤ 3 cm	31	123	0.5 ≤ CTR<1.0
Hattori(2) [17]	2016	Retrospective	IA	≤2 cm	83	270	CTR ≥ 0.5
					54	87	0.5 ≤ CTR<1.0
					29	183	CTR = 1.0
Koike [19]	2016	Retrospective	IA	≤2 cm	87	87	CTR = 1.0
Tsubokawa [20]	2018	Retrospective	IA	≤2 cm	52	44	CTR = 1.0
Tsutani [21]	2014	Retrospective	IA	≤3 cm	41	286	CTR ≥ 0.5
					28	154	0.5 ≤ CTR<1.0
					13	132	CTR = 1.0
Handa [22]	2017	Retrospective	IA	≤3 cm	29	105	>0.5

**Fig. 2:**
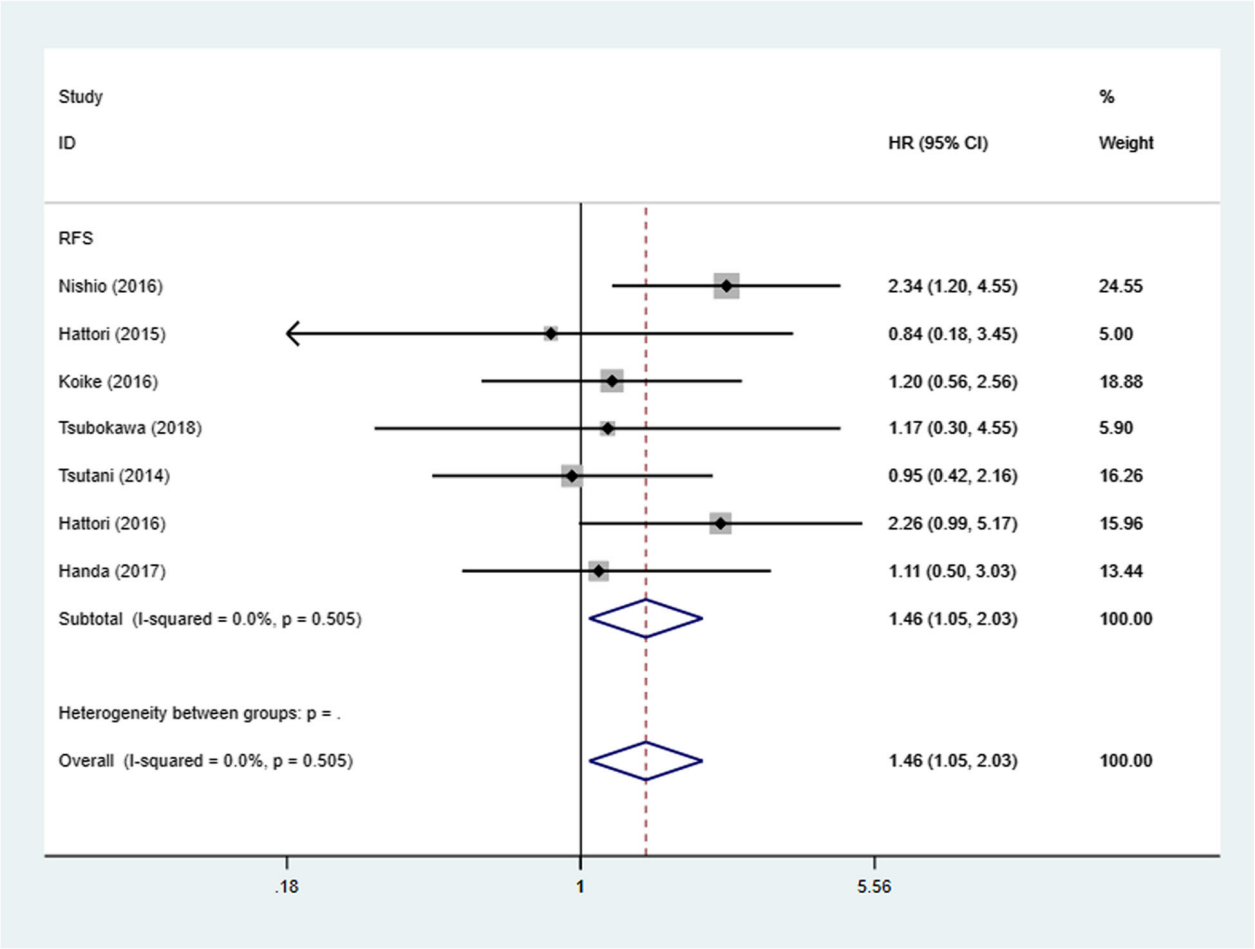
Forest plot of HR for RFS among the 7 included studies

There were 7 studies included in the RFS analysis of segmentectomy versus lobectomy in radiologically pure solid or solid dominance stage IA NSCLC [16–24]. Because there was no statistically significant heterogeneity among the studies (I^2^ = 0%, *P* = 0.505), a fixed-effects model was used to pool the hazard ratios of the studies. The pooled HR was 1.46 (95% CI, 1.05–2.03; *P* = 0.024) (Fig. 2). As the figure demonstrates, patients in the segmentectomy group had a risk of recurrence 1.46 times greater than those in the lobectomy group. In the subgroup of patients with stage IA (≤2 cm), patients treated with segmentectomy had a risk of recurrence 1.8 times greater than those treated with lobectomy (HR = 1.8; 95% CI, 1.19–2.70, *P* = 0.005). However, no significant differences were found between the segmentectomy and lobectomy groups in the pure solid subgroup (HR1.37; 95% CI, 0.82–2.29; *P* = 0.233).

**Fig. 3:**
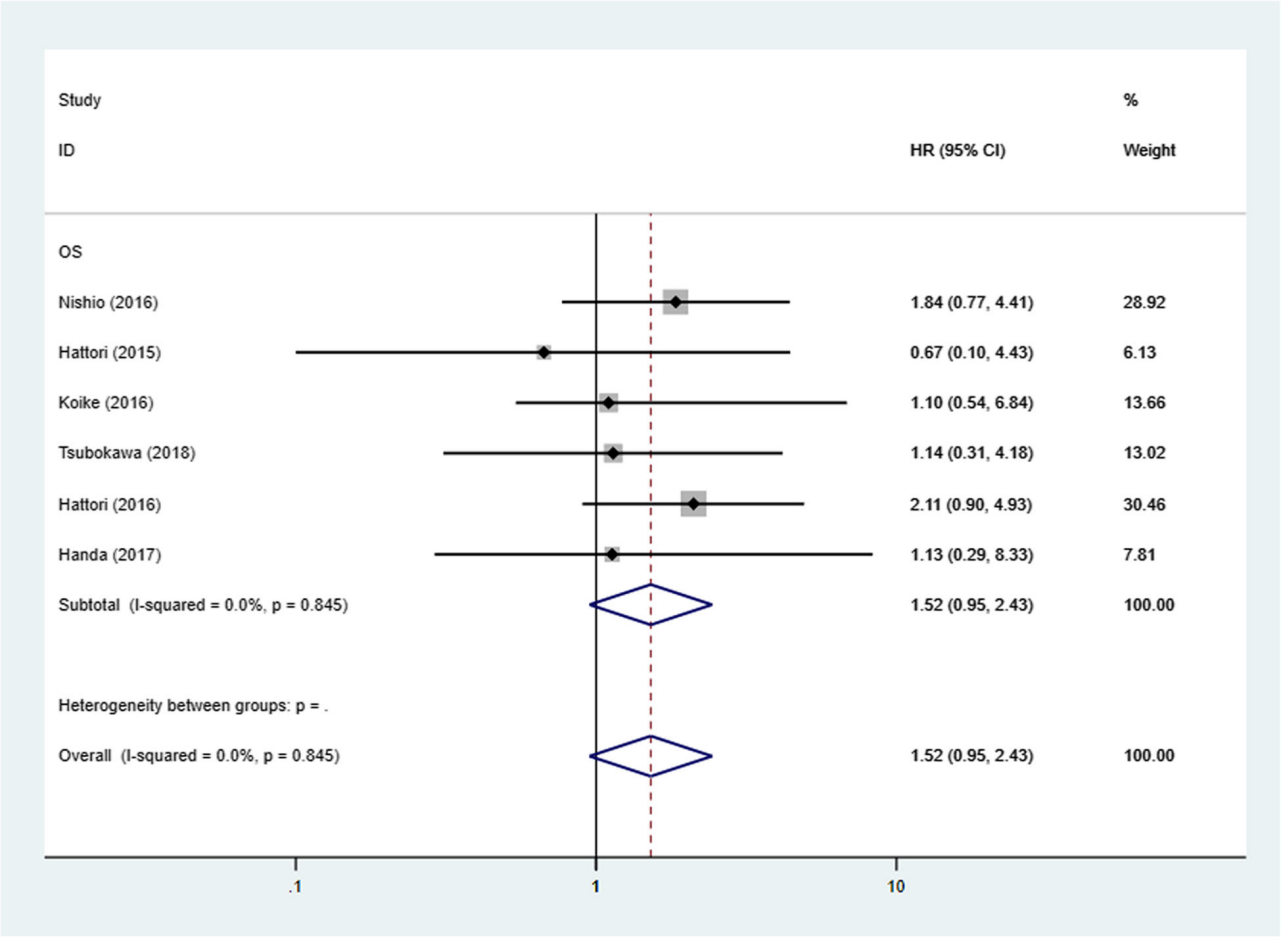
Forest plot of HR for OS among the 6 included studies

**Fig. 4:**
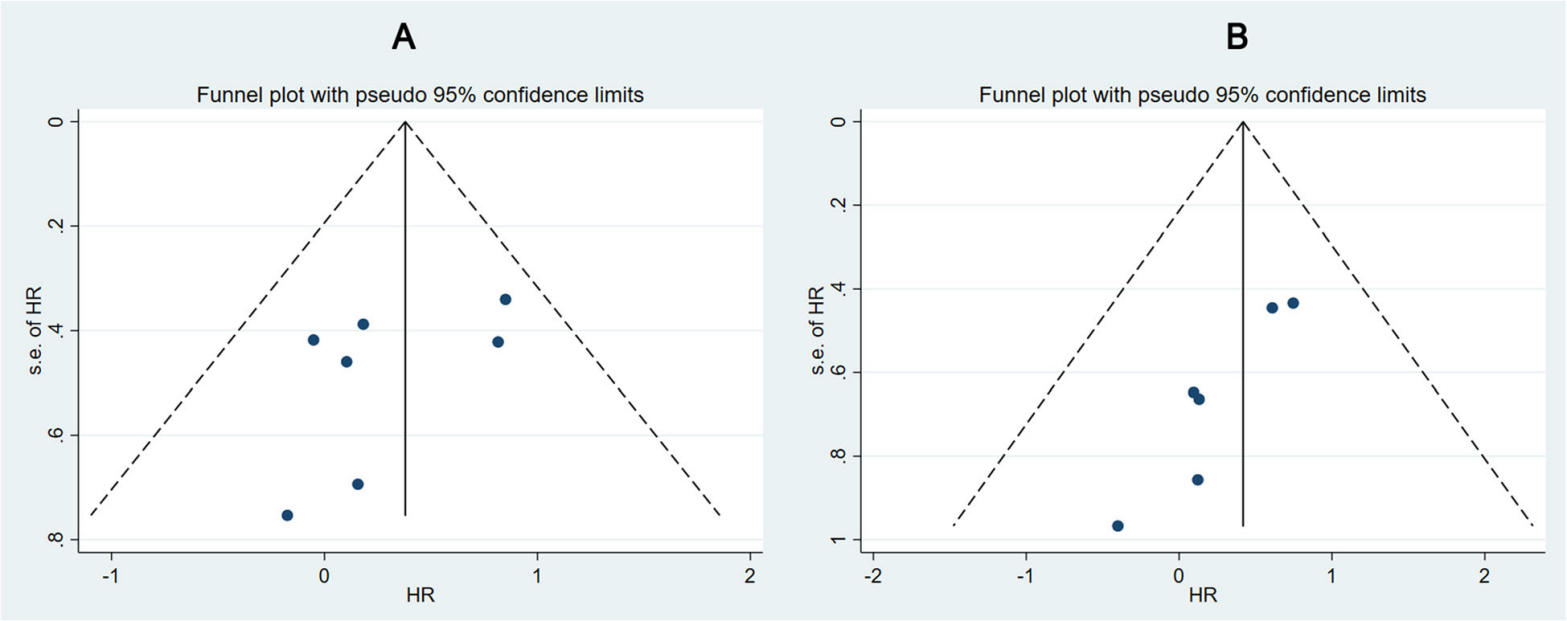
**a** presents funnel plot of HR for RFS and **b** presents funnel plot of HR for OS of stage IA NSCLC

For overall survival, 6 studies [16–20, 22] were eligible for inclusion in the analysis. The combined HR for these 6 studies was 1.52 (95% CI, 0.95–2.43; *P* = 0.08), and a fixed-effects model was applied (I^2^ = 0%, *P* = 0.845), meaning that there was no significant difference between the segmentectomy and lobectomy groups (Fig. 3). The HRs for the pure solid subgroup (HR = 1.57; 95% CI, 0.84–2.92, *P* = 0.155) and for patients with stage IA (≤2 cm) were not significant (HR = 1.66; 95% CI, 1.00–2.75, *P* = 0.051). The funnel plots for RFS did not provide any evidence of obvious publication bias and no significant publication bias was detected based on Egger’s test (*P* = 0.228). However, publication bias was found in the OS analysis (*P* = 0.005) (Fig. 4).

## Discussion

It is well established that GGO-dominant early lung NSCLC is associated with a good prognosis and that patients with these tumors are considered feasible candidates for limited surgical resection. However, radiologically determined solid-dominant tumors have a higher malignant potential than GGO-dominant tumors [12]. At present, the use of segmentectomy for solid-dominant tumors as a radical procedure is controversial. Two randomized clinical trials evaluating sublobar resection for the treatment of solid lung cancer are ongoing in Japan and the United States [26, 27]. However, it is not clear whether these two trials will determine the role of segmentectomy for solid nodules because the clinical trial in Japan included tumors with GGO, and the other trial retains the sublobar treatment arm as the comparator to lobectomy rather than subdividing the patients into two groups of segmentectomy and wedge resection.

Most previous studies believe that segmentectomy can obtain similar survival outcomes as lobectomy even in stage IA NSCLC patients with a solid-dominant or pure solid appearance on radiology. However, we made efforts to extract all detailed information to perform a comprehensive meta-analysis of lobectomy and segmentectomy for radiologically pure solid or solid-dominant stage IA non-small cell lung cancer and suggested a significant benefit of lobectomy over segmentectomy in terms of RFS in these patients. As Fig. 1 demonstrates, patients in the segmentectomy group had a risk of recurrence 1.46 times greater than those in the lobectomy group. Furthermore, the hazard ratio (HR) of RFS was higher in the subgroup of patients with stage IA (≤2 cm). In other words, lobectomy applied to a solid-dominant or pure solid tumor less than 2 cm in size had a greater survival advantage than segmentectomy. Two studies [18, 21] suggested that lobectomy had slightly worse survival outcomes than segmentectomy when the tumor was larger than 2 cm. One possible explanation for the inferior outcomes after lobectomy in these two studies was that distant recurrence was the first recurrence in many of these patients with solid-dominant or pure solid tumors larger than 2 cm, and even if the tumor was locally controlled by lobectomy, the survival outcomes for these patients may be poor because of distant recurrence. Solid or micropapillary predominant adenocarcinoma, which may present radiographically as a solid tumor, was associated with a significantly poor prognosis [28, 29]. This finding may also be why the advantage of lobectomy was reduced in the pure solid subgroup analysis. The same results were reported in these articles [19, 30].

Lobar-segmental lymph nodes (LSNs) located in a different segmental bronchus or isolated from the involved segmental bronchus were defined as the isolated lobar-segmental lymph nodes (iLSNs) by Matsumura [31]. The iLSNs are located distal to the resected segmental bronchus, and iLSNs can be difficult to resect for anatomical reasons. The advantage of lobectomy over segmentectomy is the ability to clear these nodes (iLSNs).

The status of iLSNs is crucial to determining the suitability of segmentectomy. The results in this study [31] demonstrated that 9 of 307 patients (3%) with peripheral cT1aN0M0 NSCLC had iLSN metastases. In other relevant studies [32, 33], the rate of segmental lymph node metastasis in nonprimary tumor-bearing segments (NTBSs) was 5.0% in patients with tumors smaller than 2 cm, and there was non-tumor-bearing segment metastasis even in patients with tumors less than 1 cm. When we apply segmentectomy to early lung cancers, we must avoid the possibility of recurrence in the residual pulmonary segments as much as possible.

Matsumura did not observe any patients who had solitary iLSN metastases and believes that if mediastinal-hilar nodes and the segmental lymph nodes adjacent to the involved segmental bronchus (aLSNs) are intraoperatively negative, then anatomical segmentectomy for complete resection can be performed without missing any metastatic lymph nodes. However, another study [32] reported that if the intraoperative evaluations for metastases in the lobar-hilar and mediastinal lymph nodes are performed for all patients, 1 (1.5%) in 67 patients would still have remnant tumors by segmentectomy. Therefore, determining the surgical procedure solely on the basis of intraoperative evaluation of the lobar-hilar, mediastinal lymph nodes and aLSNs there is still not an adequate method. However, systematic hilum and mediastinal lymph node dissections should be mandatory in segmentectomy and can help identify the majority of patients with intrapulmonary lymph node metastases.

Tsutani and colleagues [34] reported that the node-negative predictive criteria of solid tumors in patients with clinical stage IA lung adenocarcinoma were a size less than 0.8 cm or a maximum standardized uptake value (SUVmax) less than 1.5, which may be helpful for avoiding the risk of locoregional recurrence after limited surgical resection. In addition, several other studies [35, 36] showed that the SUVmax of the primary tumor was a prognostic factor for patients with adenocarcinoma, but not for patients with squamous cell carcinoma of the lung. On the other hand, some studies revealed that a SUVmax value > 5 was a significant predictor of lymphatic metastasis, and limited surgical resection may lead to poor prognoses in these patients. At present, there is no research showing the relationship between the SUVmax value and the status of iLSNs.

There are several limitations in the present study, and the results of the meta-analysis should be interpreted with caution. First, all of the include studies were retrospectively designed, and the clinical features of the two groups of patients were not well balanced. Although advanced statistical methods were applied in the form of propensity-score matching to balance the covariates among the arms, most studies did not consider some potential risk factors, such as the SUVmax and carcinoembryonic antigen (CEA) level. There was already a tendency to perform lobectomy for patients with large tumor diameters, large CTR values and a large extent of lymph node dissection. Second, none of the included studies provided data of on the postoperative adjuvant therapies in detail, such as chemotherapy and targeted therapy, which might affect the survival outcomes of some lung cancer patients in some way; although, it would be rare for patients with stage IA NSCLC to receive these therapies. Third, due to the lack of original data regarding the HR and associated 95% confidence interval (CI), these data obtained from the Kaplan–Meier survival curve using Parmar and Tierney’s techniques [23–25] may have contained some inaccuracies. Finally, short follow-up periods were a common problem in many studies. Cancer recurrence in this report [37] occurred up until 67 months after the operation, and death from all causes was noted up to 87 months postoperatively. Therefore, Kodama and colleagues suggested that a 10-year follow-up is appropriate to analyze the oncologic outcomes after surgical intervention for these small tumors.

Despite these limitations, we believe that our findings address important issues regarding future clinical trials for lung cancer surgery. Further studies are warranted to establish the appropriate operative strategies for radiologically pure solid or solid-dominant stage IA non-small cell lung cancer patients in a prospective setting.

## Conclusion

In conclusion, radiologically determined pure-solid or solid-dominant NSCLC tumors, even the tumors small in size, have high malignant potential; segmentectomy should not be performed for patients with radiologically determined pure-solid or solid-dominant NSCLC tumors small in size. Lobectomy is significantly associated with a better prognosis than segmentectomy in these patients. On the other hand, not all NSCLCs presenting as a solid nodule are highly invasive or easily metastasized, and the most appropriate indications for segmentectomy with a curative intent is a combination of tumor diameter, large CTR values, SUVmax, carcinoembryonic antigen (CEA) level, etc. These results will be validated by large-scale, prospective, randomized trials.

## Data Availability

The datasets generated and analyzed during the current study are available from the corresponding author on reasonable request.

## Abbreviations

aLSNs: the segmental lymph node adjacent to the involved segmental bronchus
CEA: Carcinoembryonic antigen
CI: Confidence interval
CTR: Consolidation/tumor ratio
GGO: Ground-glass opacity
HR: Hazard ratio
HSCT: Thin-section computed tomography
iLSNs: isolated lobar-segmental lymph nodes
LSNs: Lobar-segmental lymph nodes
NSCLC: Non-small cell lung cancer
NTBS: Non-primary tumor-bearing segments
OS: Overall survival
RFS: Relapse-free survival
